# Low-level laser therapy combined with scleral graft transplantation in the treatment of contracted socket: a clinical study

**DOI:** 10.1186/s12886-023-03242-3

**Published:** 2023-12-04

**Authors:** Qin Huang, Yangbin Fang, Yao Lai, Hongfei Liao

**Affiliations:** Eye Hospital of Nanchang University, Jiangxi Research Institute of Ophthalmology and Visual Science, Jiangxi Provincial Key Laboratory for Ophthalmology, Nanchang, Jiangxi China

**Keywords:** Low-level laser therapy, Vascularisation, Contracted socket

## Abstract

**Objective:**

To analyse the efficacy of the therapeutic use of low-level laser therapy (LLLT) on the tissue repair process of allogeneic scleral grafts in patients with contracted sockets by analysing the speed of graft vascularisation and fornice depth of contraction percentage.

**Methods:**

A retrospective chart review was performed from April 2015 to April 2021 including 39patients with socket contraction. Allogeneic scleral grafts were used to repair the sockets in all patients. They were randomly enrolled into two groups. The laser group included 18 patients treated with LLLT after the surgery, whereas the control group included 21 patients without LLLT after the surgery who healed naturally. The LLLT equipment used in the research had a wavelength of 650 nm, 10 mW power, and 3.8 J/cm^2^ dosimetry, and the procedure was performed once daily for 5 min over 7 days, beginning 1 week postoperatively. All patients were followed up over 6 months to examine the changes in the size of the area of the non-vascularised graft and upper and inferior fornice depth.

**Results:**

The laser group presented a significantly increased speed of conjunctival vascularisation compared with the control group (*P* = 0.003). The fornice depth of contraction percentage was more apparent in the control group than that in the laser group (*P* = 0.000).

**Conclusion:**

LLLT accelerates conjunctival vascularisation, stimulates conjunctival incision healing within a short period, shortens the tissue repair process, reduces the local inflammatory response, and causes no significant shrinkage of the conjunctival sac.

## Introduction

A sufficiently large conjunctival sac and a deep fornix are essential conditions for placing a well-fitted eye prosthesis. In some patients, years of continuous damage due to inappropriate prosthesis or orbital implant exposure can result in fornix shrinkage and progressive contraction of the socket, which will lead to severely impaired function and poor cosmetic results that would negatively affect the psychological state of those patients. Moderate to severely contracted sockets are highly challenging to treat for ophthalmologic plastic surgeons; they take a long time to treat and have slow recovery and a low success rate.

Numerous procedures have been proposed for treating contracted sockets. Forniceal reconstruction includes grafts (skin, mucous membrane, hard palate, and dermis fat) [1–5] and flaps [6–9]. The disadvantages of these autologous tissues include the limited amount of available graft material, the need for a second surgical donor site, increased operating room time, and discomfort at donor sites.

To the best of our knowledge, allogeneic scleral grafts have been used for early orbital reconstruction [10]. The advantages of this tissue are ease of access, that is, close proximity to the eye, and suitable thickness of material to sufficiently cover the area of the defect. However, allogeneic scleral grafts are at risk of dissolving [11], and measures need to be taken to vascularise the grafts as early as possible. Several studies have verified the positive outcomes of low-level laser therapy (LLLT), including promotion of wound healing [12], reduction of inflammation [13], and increased blood flow to local blood vessels [14, 15].

In this study, allogeneic sclera was used as a graft to repair the contracted socket in all patients, some of whom were treated with LLLT after surgery. We aimed to observe and compare the conjunctival vascularisation and contraction that occurred in each group over a 6-month postoperative period.

### Patients and grouping

This was a retrospective interventional case series containing data collected between April 2015 and April 2021, which included 39 patients with contracted sockets. According to the degree of conjunctival stenosis [16], all patients were diagnosed with grade 3 or 4 conjunctival stenosis and complained about the inability to retain prosthesis. There were 18 cases of simple conjunctival sac stenosis, 13 of anophthalmic socket stenosis, and eight of orbital implant exposure. The study included 26 men and 13 women, aged 13–61 years, with an average age of 39.15 years. There were 24 cases in the left eye and 15 cases in the right eye. All postoperative patients were required to do LLLT and the signing of the consent form,The LLLT required an additional 7 days and cost for treatment, so some patients refused.According to the whether the patients received the LLT after the surgery, the patients who received LLLT were included in the laser group (*n* = 18), and those who did not receive LLLT were included in the control group (*n* = 21). One week postoperatively, patients in the laser group began to receive LLLT, whereas those in the control group received no special treatment.

### Preparation and processing of allogeneic sclera material:

**Fig. 1:**
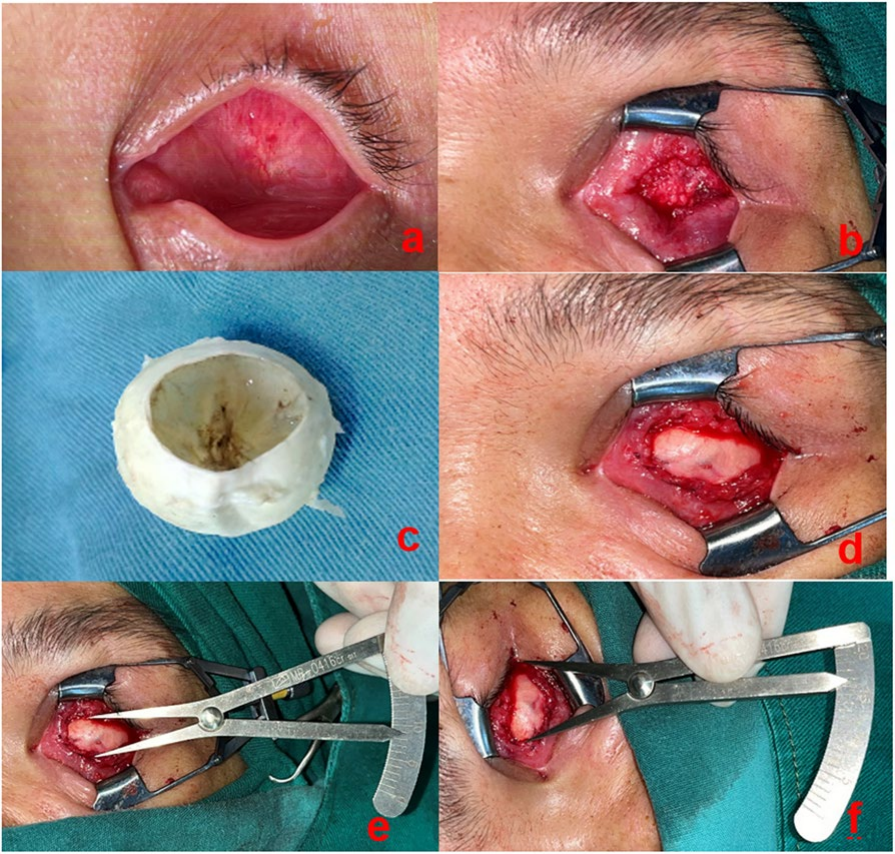
Photographs of the surgical procedure. **a** The contracted socket; **b** the cavity provided for the allogeneic sclera implant; **c** the allogeneic sclera prepared; **d** interrupted suturing of the scleral patch graft and conjunctiva in situ around the cavity; **e** a scale measuring the maximum longitudinal diameters of the scleral patch graft; and (**f**) measurement of the maximum transverse diameters

The use of allogeneic sclera material was approved by the Institutional Ethics Committee of Nanchang University and informed written consent was obtained from each patient recruited for the study in accordance with the Declaration of Helsinki. The allogeneic sclera was obtained from the eye bank of the Affiliated Eye Hospital of Nanchang University. Infectious disease indices of the donors were normal before surgery. After removing the cornea, the extrascleral tissue was removed, and the choroid was cleared. The sclera tissue was stored in a refrigerator with 95% ethanol and a low temperature of 4 °C. The sclera was removed and immersed in tobramycin saline solution for at least 2 h before the surgery to soften the sclera and was used for backup [17] (Fig. 1c).

### Surgical methods

In this study, all surgical procedures were performed under general anaesthesia by a single experienced surgeon in the same setting. The conjunctiva was opened horizontally in the centre of the socket and undermined using scissors until the superior and lower fornix relaxed or the scar tissue and contracted tissues were removed until there was no tension on the socket. The fornix was separated upward to grasp the depth, avoiding injury to the upper eyelid muscle, and downward to the lower orbital margin, creating a space at the centre of the socket and preparing the receptor bed for the graft (Fig. 1a, b). An appropriately sized plastic conformer was placed on the socket surface to observe whether the conjunctival sac was fully separated (the eyelid could naturally close as the standard). The size of the conjunctival defect was measured using a ruler and the corresponding full-thickness allogeneic scleral graft was placed in the socket over the exposed central area (Fig. 1d). The conjunctival and allogeneic scleras were sutured intermittently using 6–0 vicryl, and the edge of the allogeneic sclera was placed under the residual conjunctival surface. If the patient with conjunctival sac stenosis has no eyeball and eye socket is stable, hydroxyapatite (HA) can be implanted simultaneously. HA is a commonly used filling material and compensate orbital volume deficiency, reduce the risk of socket contraction complication, relatively light weight implants avoiding pressure over the lower lids, decreasing the risk of lower eyelid sag and upper lid sulcus deformity. The porous structure of HA is beneficial for the growth of vascular fibers and rapid vascularization is the basis of successful orbital implantation. The residual conjunctiva and fascia at the site of the plant bed provided vital vascular support to the scleral patch and anterior implant. Postoperatively, a snugly fitting conformer with multiple holes was placed in the reconstructed socket to maintain adequate pressure on the graft and the area of the fornices. The patient was bandaged for 48 h and instructed to apply ice packs for the first 24 h postoperatively and local antibiotic eye drops were used for 2 weeks.

### Laser probe and irradiation procedure

LLLT equipment (JAM2-II type; Jiangxi Teli Anesthesia & Respiration Equipment Co., Ltd.) with a wavelength of 650 nm, 10 (0–20) MW power, and a dosage of 3.8 J/cm^2^ was used. During application, the pen remained perpendicular to the edge of the junction of the conjunctiva and allogeneic sclera, with a distance of 1 cm between the irradiation surface, a spot diameter of 10 mm, and a laser power density of 12.7 mW/cm^2^. Irradiation was performed once daily for 5 min over 7 days; the plastic conformer was removed before the treatment and then put on after treatment.

### Observation indicators

**Fig. 2:**
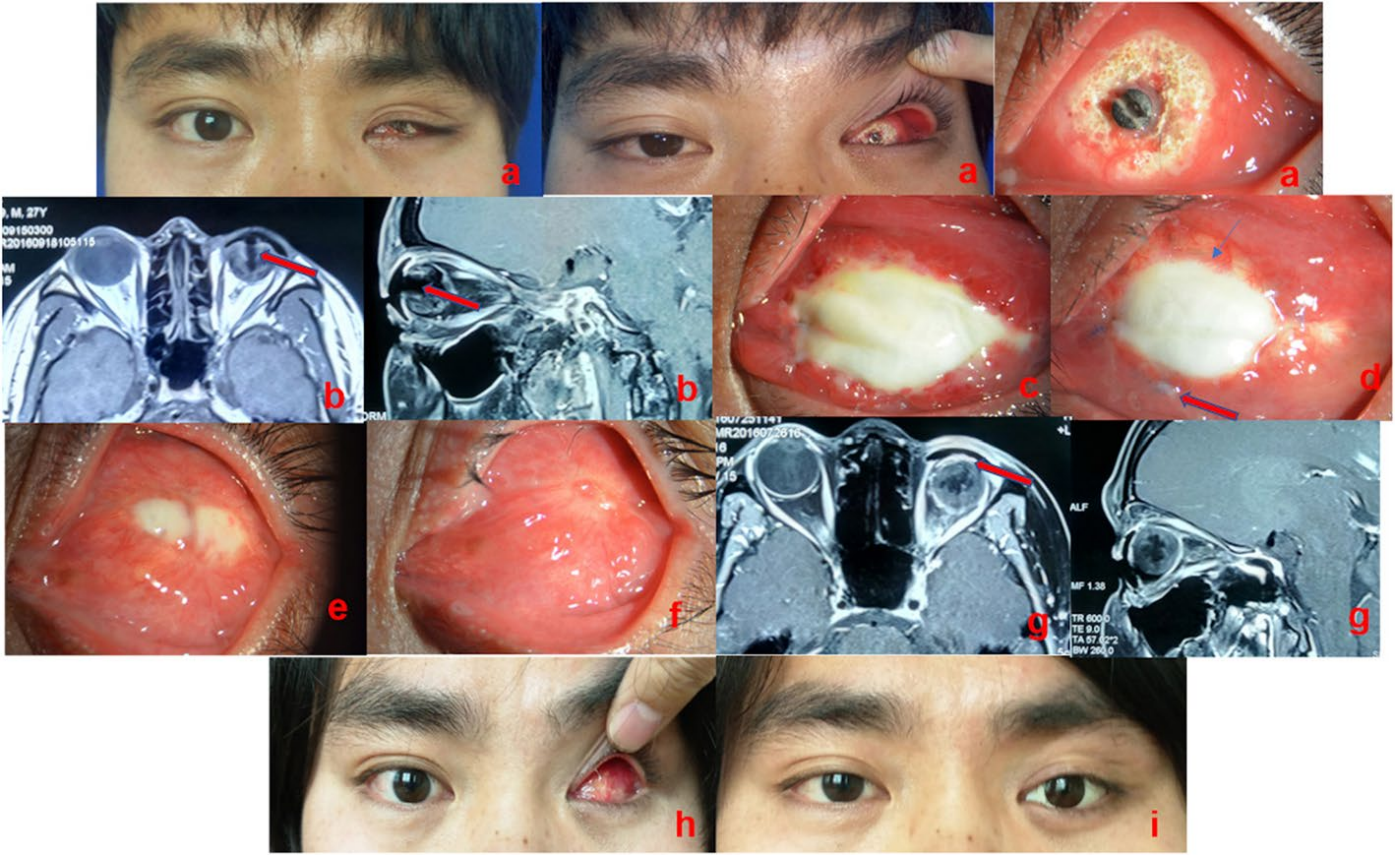
Photographs of case 1 from the laser group. **a** Patient with contracted socket combined orbital implant exposure; **b** preoperative contrast-enhanced magnetic resonance imaging (MRI): the unvascularised portion of the hydroxyapatite (HA) (red arrow); **c** anterior segment photograph: 2 weeks after the surgery; **d** one month after the surgery: the residual conjunctiva grows to the surface of the allogeneic sclera and has passed the edge of the original junction (red arrow) and neovascularisation can be observed (blue arrow); **e** six weeks after the surgery; **f** three months after the surgery; and (**g**) postoperative 6-month contrast-enhanced MRI: allogeneic sclera (red arrow) and HA is vascularised; (**h**) the conjunctival sac is completely vascularised and deep fornix is formed after low-level laser therapy; and (**i**) post-treatment photograph and prosthesis fitting

**Fig. 3:**
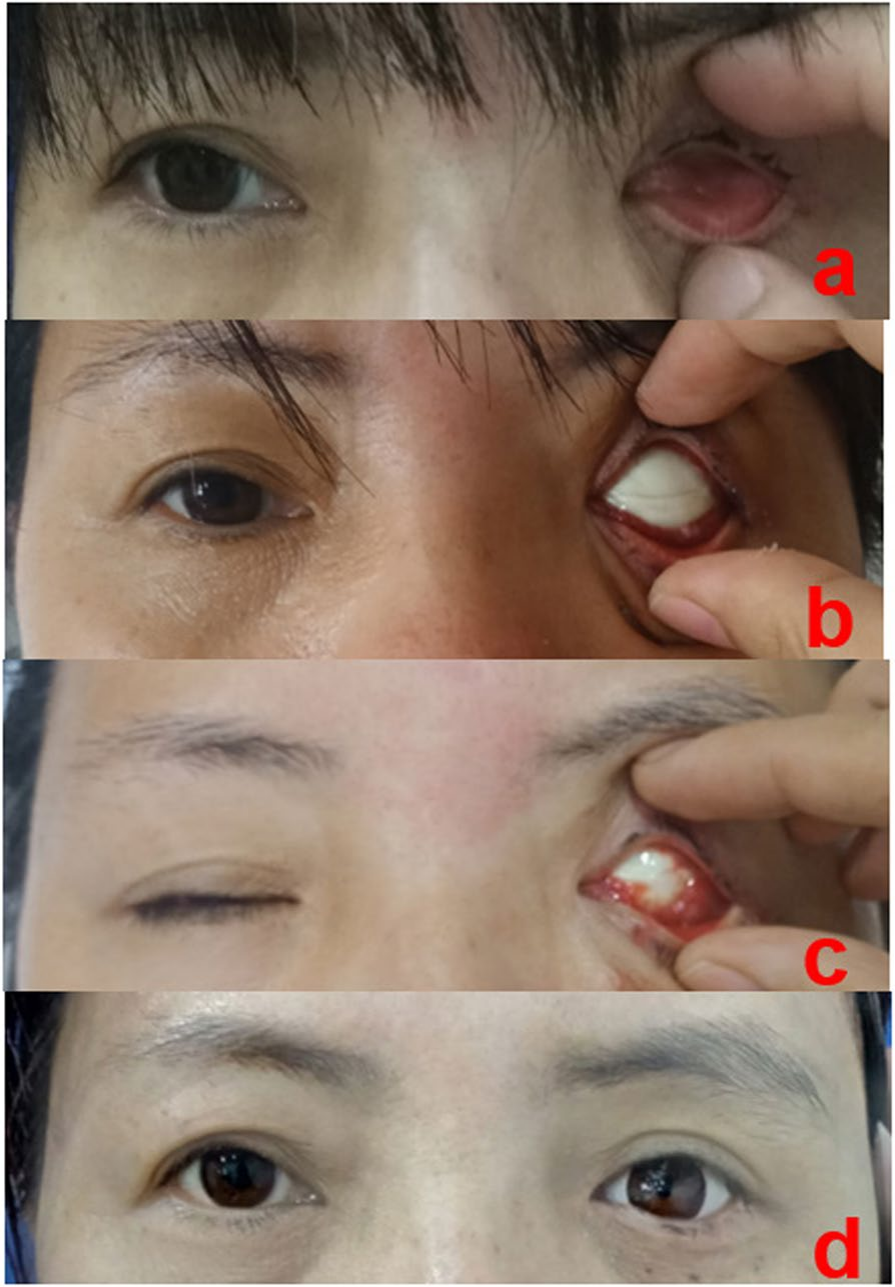
Photographs of case 2 from the laser group. **a** Patient with contracted and low-capacity anophthalmic socket; **b** one week after the surgery (hydroxyapatite implanted combined with conjunctival sac forming with allogeneic sclera covering); **c** one month after the surgery; and (**d**) prosthesis fitting

**Fig. 4:**
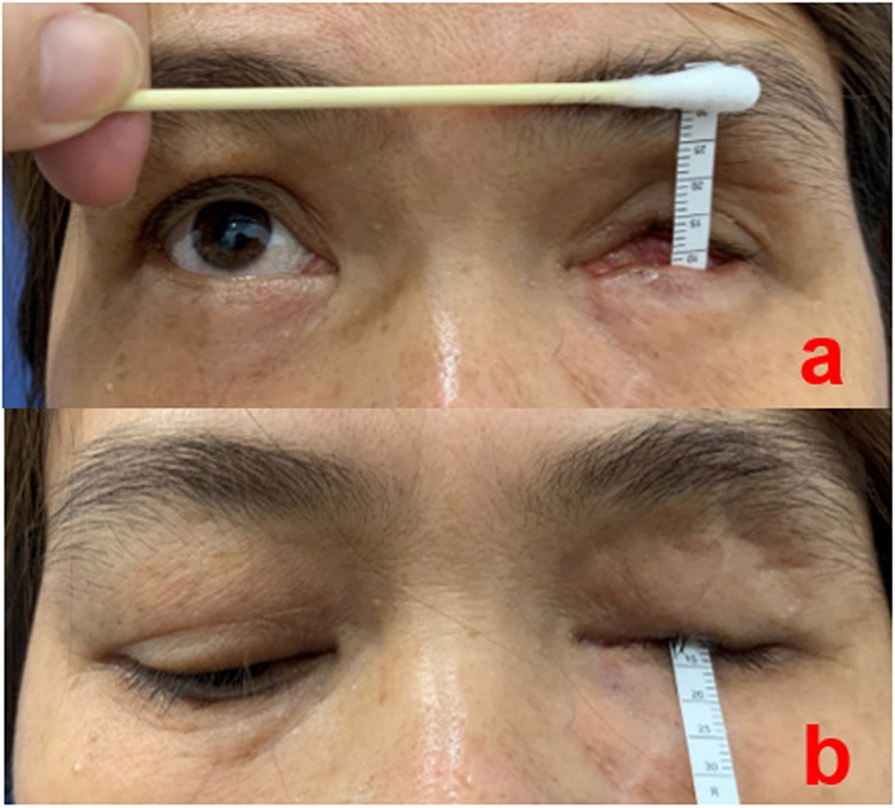
The fornices are measured by placing the scale vertically in the socket and asking the patient to look inferiorly for superior fornix and superiorly for inferior fornix. **a** Ruler positioned to obtain measurements of the inferior and (**b**) upper fornices

All postoperative data are presented at the 180-day (6-month) follow-up visit. Data were also collected immediately after the surgery and at 1, 3, and 6 months using an objective scale measuring the maximum transverse and longitudinal diameters (unit: mm) of the non-vascularised allogeneic sclera. The area of the non-vascularised graft (transverse diameter × longitudinal diameter) was calculated (Fig. 1e, f), and the speed of conjunctival vascularisation was observed (Figs. 2 and 3). Simultaneously, the fornices were measured by placing the scale vertically in the socket and asking the patient to look inferiorly for the superior fornix and superiorly for the inferior fornix. The distance between the fornix coinciding with the lid margin was recorded as the depth of the inferior fornix (IF) and superior fornix (SF) (Fig. 4), IF added to SF was the depth of the fornices. All measurements were performed by one of the authors who performed contrast-enhanced magnetic resonance imaging (MRI) 6 months postoperatively. Subjective evaluation of the presence of secretions, granulomas, or foul odours was also performed. A good outcome was defined as no prosthesis prolapse, and a poor outcome was defined as no improvement in the contracted socket that required further surgery, the inability to fit a prosthesis, or prosthesis prolapse.

## Statistical analysis

The measured data were analysed using an independent sample t-test. The therapeutic efficacy of the two groups was analysed using SPSS 22.0 statistical software. *P* < 0.05 indicated that a difference was statistically significant.

## Results

Thirty-nine patients were included in the study (Table 1.) The mean age was 36.72 ± 13.732 years in the laser group and 41.24 ± 9.818 years in the control group. There were no significant differences in age between the groups (*P* = 0.443). During the 6-month observation period, the bare area of allograft scleral grafts in 39 patients gradually decreased with time, the conjunctiva gradually grew to the scleral surface, and a new conjunctival sac was formed. All the patients could wear eye prostheses. The initial unvascularised area was 120 ± 29.126 mm^2^ in the laser group and 114.43 ± 42.859 mm^2^ in the control group, respectively; there was no significant difference in the size of the graft area between the two groups in the initial area (*P* = 0.335). One month after the surgery, we contrasted the size of the area of the non-vascularised graft, and the mean area was 42.611 ± 19.19 mm^2^ in the laser group and 68.524 ± 30.412 mm^2^ in the control group; the laser group presented a significantly increased vascularisation compared with the control group (*P* = 0.003). Three months after the surgery, the scleral graft was fully vascularized in most patients, therefore, comparisoned were not made.

**Table 1:** Anthropometric and clinical characteristics of the patients in the control and laser groups (mean ± standard deviation [SD])

	Group		Two-sided exact p^a^
	Laser group (*n* = 18)	Control group (*n* = 21)	
	Mean ± SD	Mean ± SD	
Age (years)	36.72 ± 13.732	41.24 ± 9.818	0.443
Initial unvascularised area (mm^2^)	120 ± 29.126	114.43 ± 42.859	0.335
1 month unvascularised area (mm^2^)	42.611 ± 19.19	68.524 ± 30.412	0.003
1 month growth rate (%)	65.17 ± 9.43	40.50 ± 13.11	0.000
The depth of fornices (mm)			
Immediate postoperative (mm)	22.22 ± 1.44	22.00 ± 1.10	0.587
1 month (mm)	21.94 ± 1.26	21.14 ± 0.96	0.30
3 month (mm)	21.61 ± 1.09	19.67 ± 1.06	0.00
6 month (mm)	21.06 ± 1.26	18.76 ± 0.89	0.00
Contraction percentage (%)	5.18 ± 3.01	14.62 ± 4.00	0.000

*P* < 0.05 represents significant differences

When comparing the depth of the fornices between the two groups, conjunctival sac shrinkage was more apparent in the control group than that in the laser group; the difference was insignificant in the first postoperative month (*P* = 0.30) but became significant in the third and sixth postoperative months (*P* = 0.00). The contraction percentages were 14.62 ± 4.00% and 5.18 ± 3.01%, respectively, with greater values in the control group compared with the laser group (*P* = 0.000).

In the laser group, during the follow-up period of 6 months, no obvious secretions were found in the conjunctival sac throughout the treatment process, and conjunctival sac oedema was not obvious. No significant complications were observed in the laser group resulting from laser use. However, conjunctival granuloma occurred in one patient in this group (Fig. 6). In the control group, five patients with more conjunctival sac secretions required conjunctival sac washing every week. Although the conjunctiva was completely vascularised at the final follow-up, due to inflammatory stimulation, the conjunctival grafts contracted significantly, and there was still mild conjunctival sac stenosis; thus, patients could only wear small eye prostheses.

Graft infection or foul odour was not observed in any of our patients.

## Discussion

The main causes of contracted sockets may be trauma, recurrent inflammation due to inadequate prosthesis, or radiotherapy [18]. This renders the patient unable to maintain the eye prosthesis, causing irritation and chronic discharge. If the contraction is mild, a reasonable result can be achieved by adjusting the prosthesis or deepening the fornix, such as by tightening the lower eyelid combined with fornix-forming sutures [19]. However, if the contraction is moderate to severe, repair of the contracted sockets with suitable tissues is urgently required.

Our study aimed to address various factors that contribute to contraction relapse and re-exposure of porous orbital implants; infection [20], inadequate implant vascularisation, [21] rough spicules on the implant surface [22], excessive wound tension [23], and pressure by the prosthesis on the conjunctival lining [24] have been implicated as risk factors. In recent years, the use of banked human scleral donor tissue has been increasingly scrutinised, and no confirmed cases of disease transmission via banked human scleral tissue transplantation have been reported. A scleral patch graft is inserted between the residual conjunctiva and the abrasive implant surface as a sturdy fibrous barrier that not only protects the conjunctiva from the abrasive porous implant surface but also resists melting in the early phase of hypoperfusion. The scleral patch provides a smooth surface over which the conjunctiva can simultaneously vascularise. However, the scleral patch cannot vascularise rapidly, causing psychological anxiety because patients cannot wear eye prostheses for a long time.

In this context, the tissue repair process has been the focus of several studies that have investigated treatments that increase the speed of tissue healing. LLLT is an approach that stands out for the treatment of these lesions. Studies demonstrate [25] that LLLT has been applied in wound treatment because it is effective in reducing oedema and hyperaemia in the inflammatory process, in addition to inducing the proliferation of epithelial cells, osteoblasts, and fibroblasts, favouring the synthesis of collagen. Calin and Parasca [26] found that a 630–700-nm laser can significantly repair damaged tissues. Therefore, we chose the 660-nm laser as an adjuvant treatment in our study.

Studies have shown that LLLT can increase local blood flow in tissues and promote capillary relaxation [27]. When an appropriate laser dose is used, the photon energy from LLLT can have photophysical, photochemical, and photobiological effects [28]. These effects include not only the proliferation of lymphocytes, activation of mast cells, and increase in ATP synthesis but also the proliferation of various types of cells, such as macrophages and fibroblasts. All these effects can promote anti-inflammatory and biostimulating effects, thereby promoting wound healing [15, 29].

**Fig. 5:**
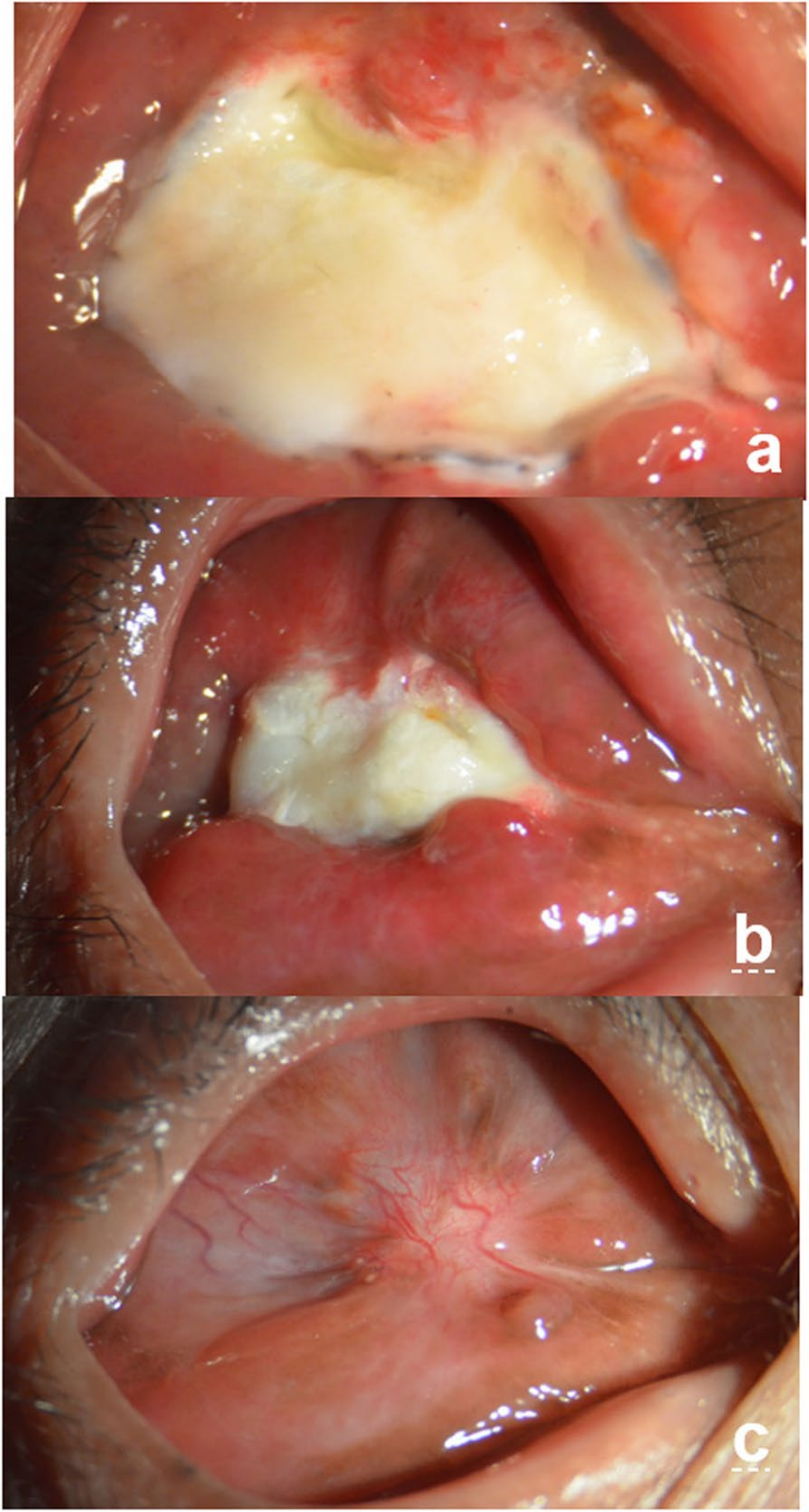
Anterior segment photograph: a patient who did not accept laser irradiation treatment. **a** The conjunctiva has more secretions, and peripheral conjunctival tissue exhibits congestion, oedema, and the allogeneic sclera with a tendency to dissolve. **b** Three months after the surgery, the peripheral conjunctival is still congested and oedematous (**c**) Six months after the surgery, the conjunctival sac is mildly contracted

Among the groups analysed in this study, the control group, which underwent conventional treatment, required more time for tissue repair process than the laser group. We observed that the presence of the conjunctiva was associated with more secretions, and the peripheral conjunctival tissue exhibited congestion, oedema, and allogeneic sclera with a tendency to dissolve (Fig. 5a, b), which may be related to the significant conjunctival sac contraction in the third and sixth postoperative months (*P* = 0.00). Mucous discharge accumulation within the socket causes chronic inflammation and fibrosis, which is consistent with the results reported by Kaltreider and Peake [30]. Due to inflammation, the conjunctival graft shrank significantly, and there was slight conjunctival sac stenosis (Fig. 5c). In contrast, we observed an effective wound treatment response in patients in the laser group over a short period, in which rapid vascularisation was visible in all studied patients. Simultaneously, good vascularisation was observed on contrast-enhanced MRI in patients with HA implantation (Fig. 2g). This was probably associated with LLLT, which contributed to the acceleration of local blood flow. Moreover, it has been demonstrated that HA orbital implants permit host fibrovascular ingrowth and reduce the risk of HA exposure [31].

**Fig. 6:**
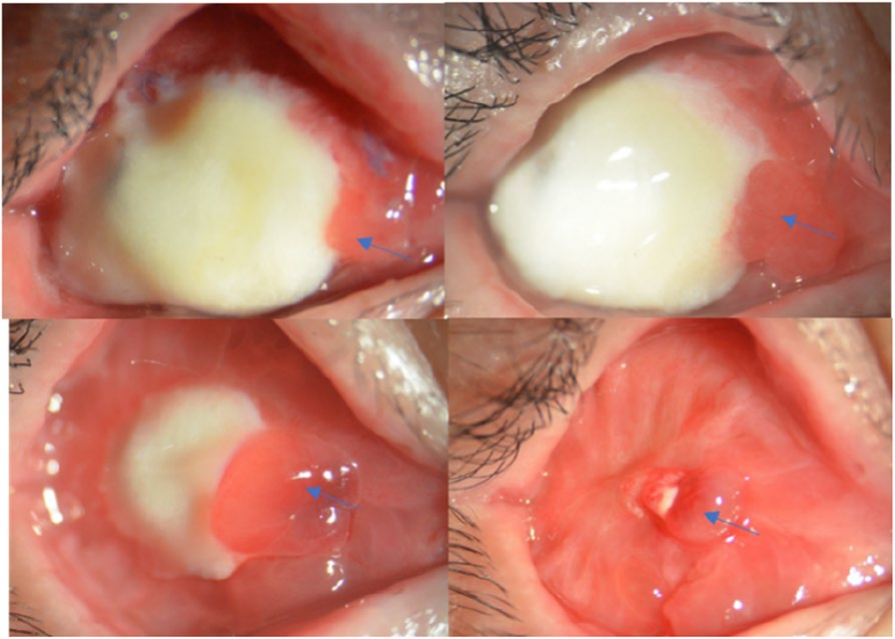
Anterior segment photograph of a patient who accepted low-level laser therapy. Conjunctival hyperplasia occurs locally, forming a granuloma (blue arrow)

Studies have shown that one of the key steps in tissue healing is the formation of granulation tissue in wounds [32]. The biological stimulatory effect generated by a semiconductor laser promotes local blood circulation, accelerates the proliferation of fibroblasts and collagen synthesis, and promotes the regeneration of epithelial cells and capillaries, thereby promoting the formation of granulation tissue in the wound. In patients who underwent LLLT in this study, we could observe that the edges of conjunctiva became active, there was an improvement in the aspect of the edges of the wound, and a minor increase of the granulation tissue. Conjunctival granuloma formation due to excessive proliferation was noted in one patient; however, no major influence was finally observed (Fig. 6).

The limitations of this study are include the small sample size and the short follow-up periods, and the laser energy and frequency were not classified owing to the limitations of the conditions, and these parameters need to be further explored.

In summary, LLLT can promote conjunctival vascularisation, shorten the course of the disease, significantly reduce the economic and psychological burden and surgical pain of patients, and reduce the risk of patients requiring reoperation. This creates favourable conditions for improving the quality of life of patients with conjunctival sac stenosis. In future clinical work, an appropriate laser wavelength, energy, and frequency can be selected for research based on the characteristics of the laser to achieve a more optimised effect.

## Data Availability

The datasets generated during and analyzed during the current study are not publicly available due to human data but are available from the corresponding author on reasonable request.
